# Statistical Research of Stainless Austenitic Steel Grain Size Distribution after Screw Rolling

**DOI:** 10.3390/ma13215048

**Published:** 2020-11-09

**Authors:** Mikhail Mikhailovich Skripalenko, Tatyana Viktorovna Zavyalova, Zbigniew Pater, Boris Alekseevich Romantsev, Stanislav Olegovich Rogachev, Liudmila Mikhailovna Kaputkina, Mikhail Nikolaevich Skripalenko, Andrei Vladimirovich Danilin

**Affiliations:** 1 Department of Metal Forming, National University of Science and Technology “MISiS”, Leninksi prospekt 4, 119049 Moscow, Russia; boralr@yandex.ru (B.A.R.); kaputkina@mail.ru (L.M.K.); tfsmn@yandex.ru (M.N.S.); danilinav@yandex.ru (A.V.D.); 2 Department of Mathematics, National University of Science and Technology “MISiS”, Leninksi prospekt 4, 119049 Moscow, Russia; tzava@yandex.ru; 3 Faculty of Mechanical Engineering, Lublin University of Technology, Str. Nadbystrzycka 36, 20-618 Lublin, Poland; z.pater@pollub.pl; 4 Department of Physical Metallurgy and Physics of Strength, National University of Science and Technology “MISiS”, Leninskiy prospekt 4, 119049 Moscow, Russia; csaap@mail.ru

**Keywords:** screw rolling, normal distribution, grain size, histogram, strain effective

## Abstract

Screw rolling of austenitic stainless-steel billets was conducted in two- and three-high mills. Statistical research results showed that, compared to heated but not rolled conditions, both screw rolling techniques provided a decrease of grain size, variance, grain size distribution asymmetry, and excess in the billet cross-section at the stationary stage of screw rolling. At that stage, grain size distribution after two-high screw rolling is closer to normal in terms of asymmetry and excess values compared to grain-size distribution after three-high screw rolling. A strong negative correlation between strain effective values and grain-size values for the cross-section of the rolled billets at the stationary stage was revealed for both two- and three-high screw rolling.

## 1. Introduction

Grain structure and, essentially grain boundaries, clearly define steel mechanical properties. In particular, the Hall–Petch relationship connects the yield point with the grain size (the distance between the grain boundaries d):

(1) $$\sigma_{0,2}=\sigma_{0}+K_{y}d^{-\frac{1}{2}}$$

where σ_0_ is the friction stress connected with the sliding of dislocations in a monocrystal, and K_y_ is the material’s constant. It is very rare to obtain material with grains (crystals) equal in size and shape. The so-called uniform grain structure (or close to such) occurs in practice for most cases, and such a structure is considered when grain size distribution is close to normal. It could also be nonuniform anisomerous, which can be introduced as a whole (sum) of several normal distributions.

After primary recrystallization, the structure of alloys usually serves as an example of the first type of structure given in the previous paragraph. The structure obtained after secondary recrystallization [1] or partial recrystallization while heating or forming [2] could be an example of the second type of structure given in the previous paragraph. The orientational dependence of interfacial distances is important for structured anisotropic materials, which are metals and alloys after forming.

Nonuniform grain structure may appear at nonuniform deformation or temperature through the investigated object’s volume or section. It may also appear as complex, for example, the spiral shape of grains, and when the nonuniform structure depends on the section’s orientation. It is necessary to specify how, including the studied metallographic section’s orientation, the structure’s image was obtained while comparing grain structures of different objects in any case. All structure-dependent properties are defined by morphology, dimensions, and the distribution of different structural elements through volume. For monophase polycrystalline materials, grain (crystallite) is the item which governs choosing thermo-deformation treatment. Grain size value and its distribution through billets volume are more definitive for mechanical properties. Grain boundary interactions with alloying elements and impurities sufficiently influence an alloys’ corrosion resistance. At the same time, grain size is quite easy to measure. This is why grain size may be used for the estimation of an obtained metal structure’s quality, and statistical criteria of grain size value distribution and changing of these criteria because of influence of different factors may be used for the investigation of different structure formation processes (recrystallization, grain growth and others).

If there is no aim to obtain gradient structure and properties material condition, then uniform structure is more preferable, due to, at least, two items: properties are easier to forecast and control; a high level of special properties, for instance, corrosion resistance, is achieved more often.

Two-high [3,4] and three-high [5,6] screw rolling mills are used in metallurgical manufacturing. Screw rolling at high values of feed angles (18 degrees or more) and inclination angles (10 degrees or more), the so-called radial shear rolling [7], is referred to severe plastic deformation (SPD) processes [8]. The grain structure of many materials becomes ultrafine and nanostructured after SPD processes [9,10], and, as a result, the materials’ physical and mechanical properties are changed [11,12]. Materials often have a structure with a bimodal distribution of grain size after SPD processes. For example, Kozulin et al. [13] found that an aluminum–magnesium alloy had a grain structure with a bimodal grain size distribution after equal channel angular pressing. A bimodal grain size distribution was also observed after the multi-pass hot rolling of a magnesium alloy [14], after groove pressing of an aluminum alloy [10], and after accumulative roll bonding (ARB) of copper [15]. It was also noted that the bimodal distribution of grain size was detected after the radial-shear rolling of an AZ31 magnesium alloy [16].

Considering the widespread availability of computer-aided tools for grain structure analyses, it is essential to estimate the characteristics of a grain structure formed after screw rolling using mathematical statistics techniques.

The research objective was to statistically analyze grain size (the distance between grain boundaries) distribution in the AISI 321 billets’ cross-section after two- and three-high screw rolling at an equal temperature and similar degree of strain. The aim was to reveal differences between two- and three-high screw rolling in the way they influence grain size distribution and, if possible, to establish connections between differences in the strain state with the differences in the grain size distribution.

## 2. Materials and Methods

Screw rolling of AISI 321 billets (NUST “MISiS”, Moscow, Russian Federation) was carried out in two- and three-high rolling mills. The initial billet had a diameter of 60 mm and a length of 200 mm. The billets were preliminarily heated up to 1150 °C and maintained at this temperature for 2 h. The diameter of the billets after two-high screw rolling was 54 mm; after three-high screw rolling, it was 52 mm. The billets were cooled in water after rolling. Two-high screw rolling was conducted using an MISIS-130D mill (The Electrostal Heavy Engineering Works JSC (“EZTM” JSC), Electrostal city, Moscow Region, Russian Federation) [17]; three-high screw rolling was conducted using an MISIS-130T mill (The Electrostal Heavy Engineering Works JSC (“EZTM” JSC), Electrostal city, Moscow Region, Russian Federation) [17]. The feed angle of the rolls of the MISIS-130D mill was 18 degrees, and the inclination angle of the rolls was 0 degrees. The feed angle of the MISIS-130T mill rolls was 18 degrees; the inclination angle of the rolls was 10 degrees. The rolls rotational speed was 5.76 rad/s.

**Figure 1:**
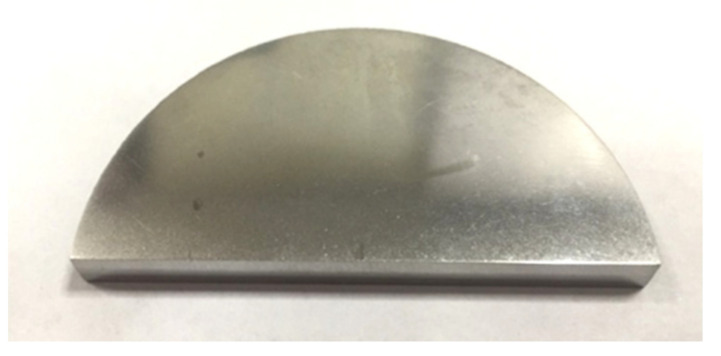
Sample with a metallographic section in the cross-section of the billet for microstructure investigation.

One semicircle sample of 5 mm thickness (Figure 1) was cut from the middle of each of the billets after forming in two-high and three-high mills to compare stationary stages of rolling. Cutting was conducted with water-cooling; the width of the cut was 0.1 mm. Metallographic sections were done for the cross-section of the samples. The grain size was measured along the billets’ radius using a linear-intercept method. The research was carried out using an Axio Observer D1m Carl Zeiss optical microscope (Carl Zeiss Microscopy GmbH, Jena, Germany), with Tixomet software, LLC “Tixomet”, St. Petersburg, Russian Federation) with ×200 magnification. One thousand two hundred measurements were performed for each of the metallographic section. The same measurements (1200 measurements) were performed for the sample cut out from the billet that was identically heated but not rolled (the reference billet).

## 3. Results

**Figure 2:**
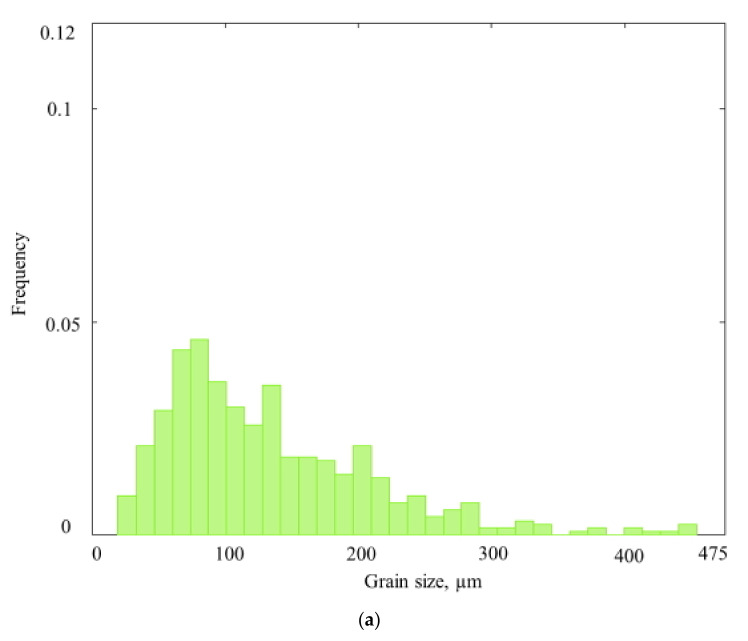

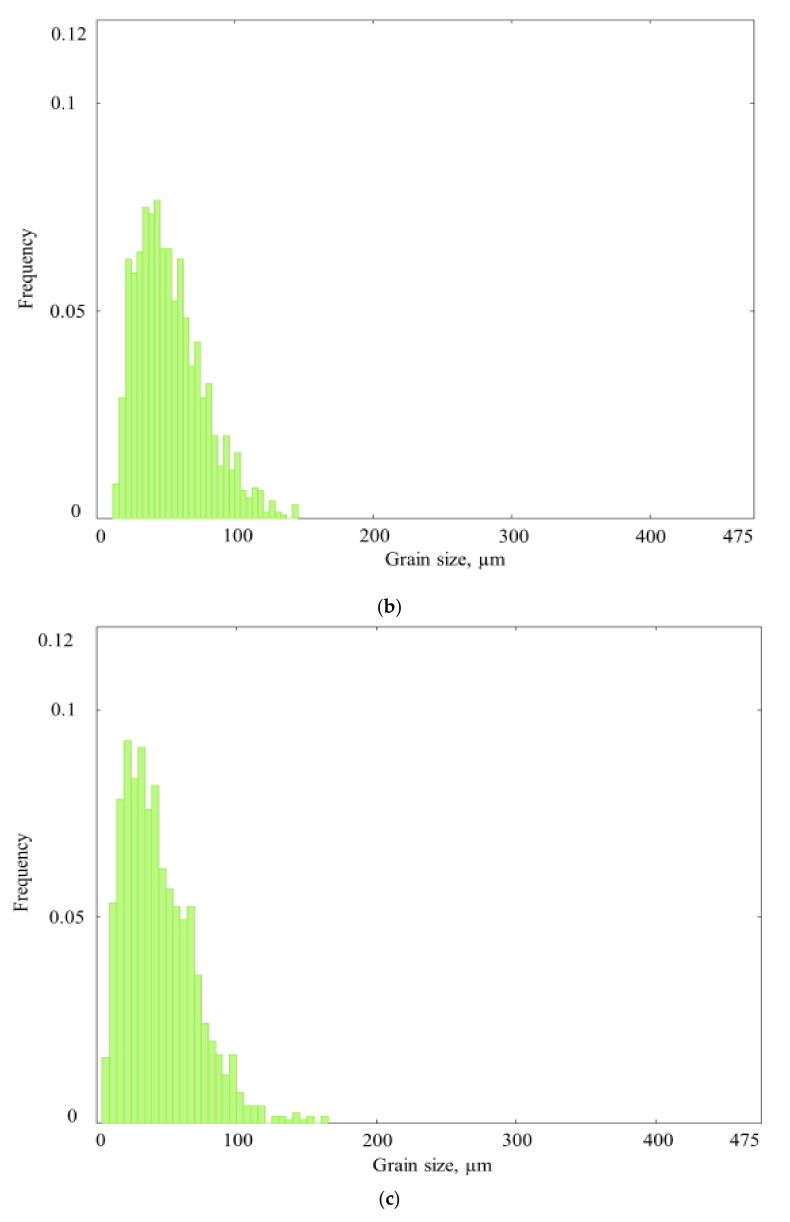
Histograms of the grain size distribution of the reference billet (**a**) after two-high screw rolling, and (**b**) three-high screw rolling (**c**).

The histograms of the austenitic grain size distribution after different types of treatment are shown in Figure 2.

**Figure 3:**
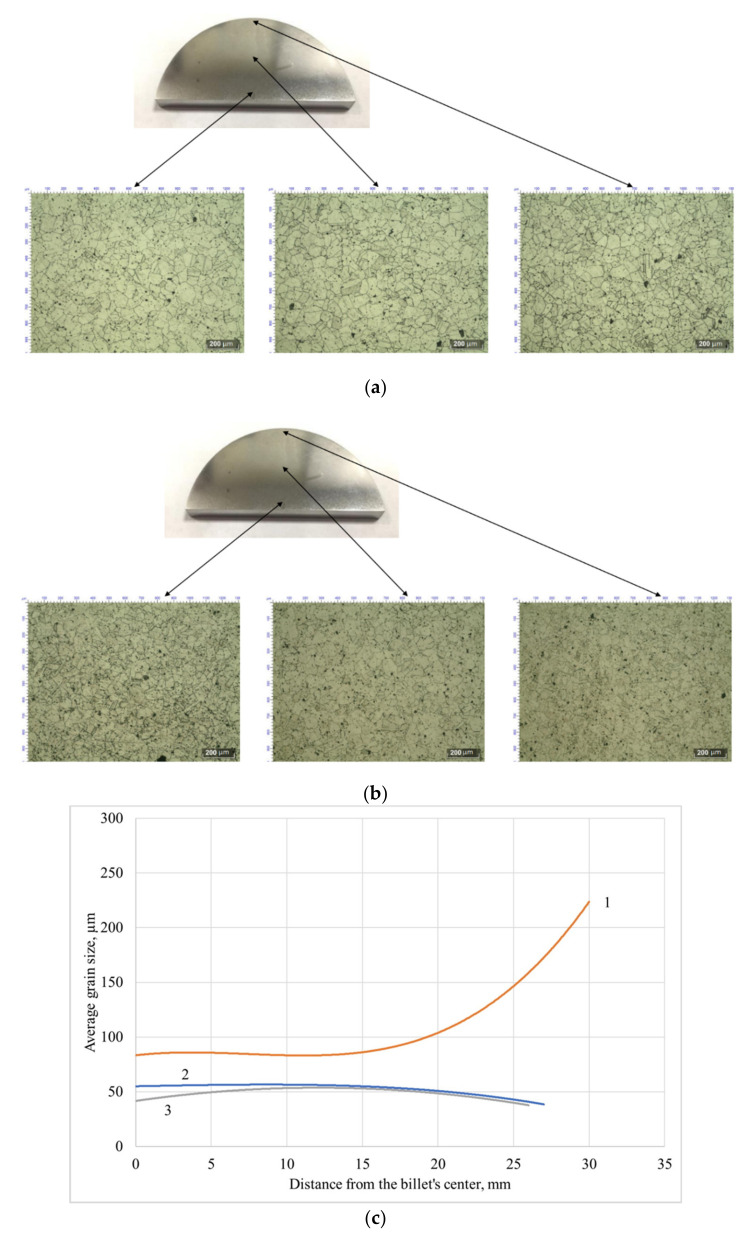
Microstructure of the billet at the stationary stage after two- (**a**) and three-high screw rolling (**b**), and change of the average grain size (**c**) of the reference billet (1), the billet after two-high screw rolling (2), and the billet after three-high screw rolling (3).

It can be seen that (Figure 2) the mean value, variance, range, and asymmetry of the grain size distribution decrease after both screw rolling techniques. The reasons for such changes are sufficient grain size decreasing, recrystallization, and, probably, changing of the grains’ shape. All these changes occurred mainly close to the rolled billet’s surface where high values of the strain rate were observed [17], which was the place in the reference billet where the grains of the largest size were located (Figure 3). If all the billet layers were rolled with equal strain and post-rolling processes passed equally (in case all other conditions being the same), then the histograms should have been the same as for the reference billet with just a change in values along the x-axis.

Asymmetry and excess of the grain size distribution, the average grain size, and the variance for each of the billets were calculated (Table 1).

It is known that asymmetry and excess values are equal to 0 for normal distributions. Positive excess, which occurred for each of the three billets, meant that there were more “outliers” in the sample than in a normal distribution, and that all three samples had kurtosis in the distribution. All the grain size distribution parameters changed after both screw rolling techniques (Table 1). Some slight differences may be observed. The asymmetry coefficient after two-high screw rolling, compared to three-high screw rolling, was less than one. This value was more than one after three-high screw rolling. According to Table 1, the excess is 8.5 times less after two-high screw rolling and almost three times less after three-high screw rolling, compared to this number for the reference billet. This meant that both screw-rolling techniques, from the statistical point of view, made the grain size distribution “closer to single mode.” A similar effect may be noticed while comparing the asymmetry coefficient values. According to Table 1, there is sufficient positive (or left-side) asymmetry for the reference billet: values less than the mean value were found most often in the sample [18]. A probable reason for this phenomenon is heating and holding of the billet, as there are large grains near the billet’s surface (Figure 3); their size influences the sample mean grain size value, although there are not many of them. The grains with smaller sizes are much more numerous, which moved the distribution to the left (Figure 2). Two-high screw rolling decreased the asymmetry by 2.1 times compared to three-high screw rolling that decreased the asymmetry by 1.7 times. This shows that two-high screw rolling, even with the diameter reduction being slightly less than that of three-high screw rolling, lessens excess (kurtosis of the distribution) and asymmetry much greater.

**Figure 4:**
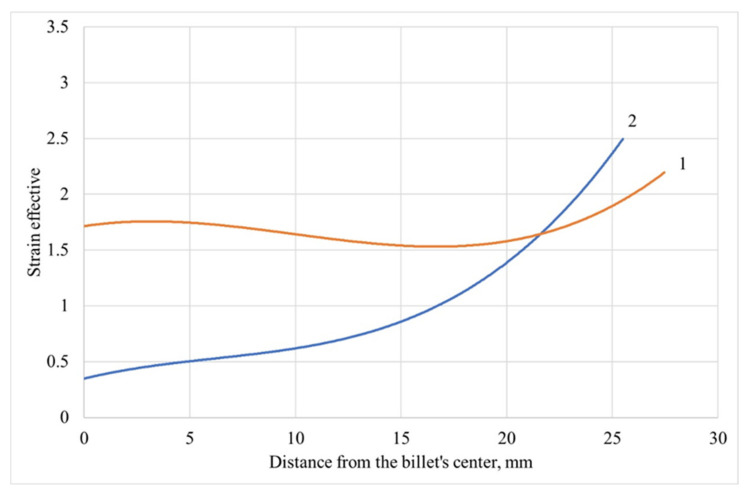
Changing of strain effectiveness in the cross-section of billet at the stationary stage of two-high (1) and three-high (2) screw rolling.

**Figure 5:**
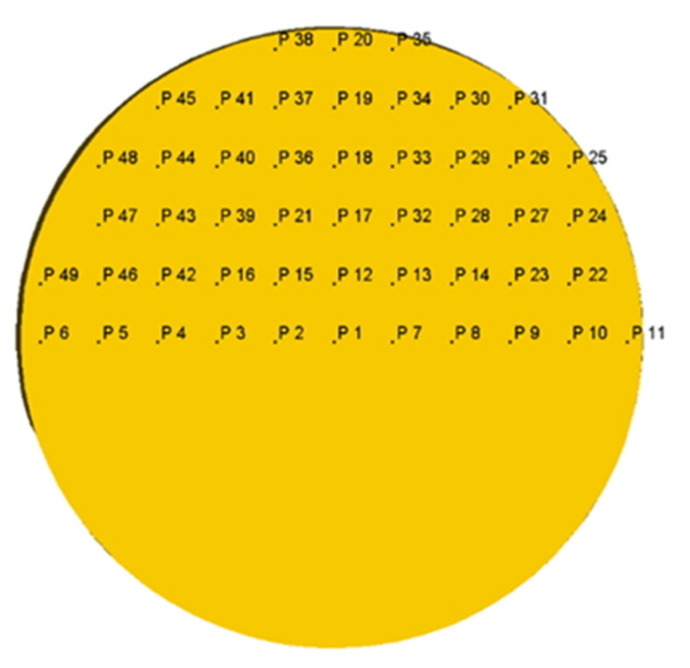
Points in the cross-section of the billet at the stationary stage of screw rolling for which the strain effective values were calculated due to computer simulation.

Computer simulations of the investigated screw rolling processes were created in [19], and the simulation allowed the calculation of strain effective:

(2) $$\bar{\varepsilon }=\frac{\sqrt{2}}{3}\sqrt{{\left(\varepsilon_{1}-\varepsilon_{2}\right)}^{2}+{\left(\varepsilon_{2}-\varepsilon_{3}\right)}^{2}+{\left(\varepsilon_{3}-\varepsilon_{1}\right)}^{2}}$$

where ε_1_, ε_2_, and ε_3_ are the principal strains. According to [19], a strong negative correlation was established between strain effective and grain size at the non-stationary stage of two- and three-high screw rolling. Using the computer simulation results [19,20], it was established that there is a difference in the penetration depth of the deformation. It is qualitatively seen from the trend lines of Figure 4, which are governed by third-grade polynomials, whose strain effective changes along the radius at two-high screw rolling are less than at three-high screw rolling. Trend lines are made for strain-effective values at the billet cross-section points at the stationary stage of screw rolling (Figure 5). The range of strain effective values for two-high screw rolling was 2, and the variance was 0.4; the range of strain effective values for three-high screw rolling was 2.5, and the variance was 0.5. A more uniform distribution of the strain effective values at two-high screw rolling allows for refining of the grain structure in the billet’s center compared to three-high screw rolling, and this could be the reason for lower asymmetry and excess of the grain size distribution: there is only one column with 0.075 frequency or more after two-high screw rolling, whereas there are five columns with 0.075 frequency or more after three-high screw rolling (Figure 2b,c). More uniform distribution of strain-effective values could lead to less grain size variance after two-high screw rolling, which equals 591.5, compared to the same after three-high screw rolling −654.3.

The correlation coefficient was calculated to estimate the influence of effective strain on the grain size formation at the stationary stage of two- and three-high screw rolling. The correlation coefficient was −0.83 for two-high screw rolling and −0.87 for three-high screw rolling. It is worth noting that effective strain could be a qualitative measure of the driving force for texture and recrystallization, however, effective strain solely does not capture the complexity of the thermo-mechanical history of screw rolling processes.

## 4. Conclusions

A similar process of the grain structure changes leading to a mean grain size value decrease, and the nonuniformity of grain size values decrease along the cross-section of the billets were observed after hot two- and three-high screw rolling of the AISI 321 steel billets at the same temperature and close degrees of strain. This is clearly reflected in the grain size distribution. The statistical investigation confirmed that, after both types of screw rolling techniques, the mean grain size, the grain-size variance, the asymmetry coefficient, and the excess of the grain size distribution decreased compared to the reference billet parameters. The grain-size distribution after two-high screw rolling was closer to normal in terms of asymmetry and excess compared to the grain size distribution after three-high screw rolling.

A strong negative correlation (the correlation coefficient was less than −0.75) was noted between effective strain and grain size at the stationary stage after both screw rolling techniques.

## Figures and Tables

**Table 1:** Characteristics of the grain size distribution for the reference billet and the billets after two- and three-high screw rolling.

Billet	Average Grain Size, µm	Variance of Grain Size, µm^2^	Asymmetry Coefficient	Excess
Reference	103	4271.9	1.84	4.61
After two-high screw rolling	53	591.5	0.86	0.54
After three-high screw rolling	45	654.3	1.08	1.55

## References

[1] Guliaev A.P., Guliaev A.A. (2012). Physical Metallurgy. University Handbook.

[2] Gorelik S.S., Dobatkin S.V., Kaputkina L.M. (2005). Recrystallization of Metals and Alloys.

[3] Pater Z., Kazanecki J. (2013). Complex Numerical Analysis of the Tube Forming Process Using Diescher Mill. Arch. Metall. Mater..

[4] Chastel Y., Diop A., Fanini S., Bouchard P., Mocellin K. (2008). Finite Element Modeling of Tube Piercing and Creation of a Crack. Int. J. Mater. Form..

[5] Stefanik A., Szota P., Mróz S., Bajor T., Boczkal S. (2016). Influence of the Deformation Method on the Microstructure Changes in AZ31 Magnesium Alloy Round Rods Obtained by the Rolling Process. Key Eng. Mater..

[6] Shatalov R.L., Medvedev V.A. (2020). Regulation of the Rolling Temperature of Blanks of Steel Vessels in a Rolling-Press Line for the Stabilization of Mechanical Properties. Metallurgist.

[7] Dobatkin S., Galkin S., Estrin Y., Serebryany V., Diez M., Martynenko N., Lukyanova E., Perezhogin V. (2019). Grain refinement, texture, and mechanical properties of a magnesium alloy after radial-shear rolling. J. Alloys Compd..

[8] Diez M., Kim H.-E., Serebryany V., Dobatkin S., Estrin Y. (2014). Improving the mechanical properties of pure magnesium by three-roll planetary milling. Mater. Sci. Eng. A.

[9] Glezer A.M., Sundeev R.V. (2015). General view of severe plastic deformation in solid state. Mater. Lett..

[10] Moskvichev E., Skripnyak V., Skripnyak V., Kozulin A., Lychagin D. (2018). Structure and Mechanical Properties of Aluminum 1560 Alloy after Severe Plastic Deformation by Groove Pressing. Phys. Mesomech..

[11] Meyers M., Mishra A., Benson D. (2006). Mechanical properties of nanocrystalline materials. Prog. Mater. Sci..

[12] Meyer L., Hockauf M., Krüger L., Schneider I. (2007). Compressive behaviour of ultrafine-grained AA6063T6 over a wide range of strains and strain rates. Int. J. Mater. Res..

[13] (2020). Mechanical Properties of Al-Mg Alloys after Severe Plastic Deformation. Contemporary Problems of Science and Education (Scientific Journal). http://www.science-education.ru/ru/article/view?id=11300.

[14] Fukuda Y., Noda M., Ito T., Suzuki K., Saito N., Chino Y. (2017). Effect of Reduction in Thickness and Rolling Conditions on Mechanical Properties and Microstructure of Rolled Mg-8Al-1Zn-1Ca Alloy. Adv. Mater. Sci. Eng..

[15] Li M., Liu J., Jiang Q. (2017). Effect of Annealing Temperature on Tensile Fracture Behavior of ARB-Cu at Room Temperature. Acta Metall. Sin..

[16] Stefanik A., Szota P., Mróz S. (2020). Analysis of the effect of rolling speed on the capability to produce bimodal-structure AZ31 alloy bars in the three-high skew rolling mill. Arch. Metall. Mater..

[17] Skripalenko M., Romantsev B., Kaputkina L., Galkin S., Skripalenko M., Cheverikin V. (2019). Study of Transient and Steady-State Stages During Two-High and Three-High Screw Rolling of a 12Kh18N10T Steel Workpiece. Metallurgist.

[18] Korn G., Korn T. (2017). Mathematical Handbook for Scientists and Engineers.

[19] Skripalenko M., Romantsev B., Galkin S., Kaputkina L., Skripalenko M. (2019). Study of Strain and Structural Peculiarities in Different Stages of Two- and Three-High Screw Rolling. Steel Transl..

[20] Skripalenko M.M., Romantsev B., Galkin S., Kaputkina L., Skripalenko M.N., Danilin A., Rogachev S. (2020). Forming Features at Screw Rolling of Austenitic Stainless-Steel Billets. J. Mater. Eng. Perform..

